# Anosmia and Ageusia as Predictive Signs of COVID-19 in Healthcare Workers in Italy: A Prospective Case-Control Study

**DOI:** 10.3390/jcm9092870

**Published:** 2020-09-04

**Authors:** Giuseppe La Torre, Anna Paola Massetti, Guido Antonelli, Caterina Fimiani, Mauro Fantini, Mattia Marte, Augusto Faticoni, Carlo Maria Previte, Ombretta Turriziani, Francesco Pugliese, Paolo Villari, Ferdinando Romano, Claudio Maria Mastroianni, Covid-19 Sapienza Collaborative Group

**Affiliations:** 1 Department of Public health and Infectious Diseases, Sapienza University of Rome, 00185 Rome, Italy; paola.massetti@uniroma1.it (A.P.M.); catfim@yahoo.com (C.F.); fantini.1640596@studenti.uniroma1.it (M.F.); mattia.marte@uniroma1.it (M.M.); augusto.faticoni@uniroma1.it (A.F.); carlo.previte@uniroma1.it (C.M.P.); paolo.villari@uniroma1.it (P.V.); ferdinando.romano@uniroma1.it (F.R.); claudio.mastroianni@uniroma1.it (C.M.M.); 2 Department of Infectious Diseases, Teaching Hospital “Policlinico Umberto I”, Sapienza University, 00185 Rome, Italy; 3 Department of Molecular Medicine, Teaching Hospital “Policlinico Umberto I”, Sapienza University, 00185 Rome, Italy; guido.antonelli@uniroma1.it (G.A.); ombretta.turriziani@uniroma1.it (O.T.); 4 Division of Anesthesiology and Intensive Care, Teaching Hospital “Policlinico Umberto I”, Sapienza University of Rome, 00185 Rome, Italy; f.pugliese@uniroma1.it; 5 Covid-19 Sapienza Collaborative Group, Sapienza University of Rome, 00185 Rome, Italy; rosario.cocchiara@uniroma1.it

**Keywords:** COVID-19, SARS-CoV-2, symptoms, signs, anosmia, ageusia, healthcare workers, Italy

## Abstract

Background: The aim of this study was to investigate the diagnostic accuracy of symptoms and signs in healthcare workers (HCW) with Sars-CoV-2. Methods: This was a case-control study. Cases consisted of symptomatic healthcare workers who had a positive SARS-CoV-2 real-time polymerase chain reaction (RT-PCR) test, while controls were symptomatic healthcare workers with a negative RT-PCR test. For each symptom, ROCs were plotted. Diagnostic accuracy was calculated using the sensitivity, specificity, and positive and negative predictive values. A logistic regression analysis was carried out for calculating the OR (95% CI) for each symptom associated to the SARS-CoV-2 positivity. Results: We recruited 30 cases and 75 controls. Fever had the best sensitivity while dyspnea, anosmia, and ageusia had the highest specificity. The highest PPVs were found again for dyspnea (75%), anosmia (73.7%), and ageusia (66.7%). Lastly, the highest NPVs were related to anosmia (81.4%) and ageusia (79.3%). Anosmia (OR = 14.75; 95% CI: 4.27–50.87), ageusia (OR = 9.18; 95% CI: 2.80–30.15), and headache (OR = 3.92; 95% CI: 1.45–10.56) are significantly associated to SARS-CoV-2 positivity. Conclusions: Anosmia and ageusia should be considered in addition to the well-established fever, cough, and dyspnea. In a resource-limited setting, this method could save time and money.

## 1. Introduction

COVID-19 is currently spreading in Italy. Positive patients present a wide range of signs and symptoms, including fever and dry cough, as well as a severe interstitial pneumonia, acute distress respiratory syndrome (ARDS), multiorgan failure, and even death [1]. Patients usually develop the first symptoms after 2–11 days up to a maximum of 14 days, although some reported cases have a longer incubation period [2].

The Italian National Health System (SSN) suffered many losses due to COVID-19 among healthcare workers (HCWs). According to the report issued on 30 March 2020 by the Istituto Superiore di Sanità (ISS) 9% (*n* = 8920) of the total reported cases (*n* = 94.312) hit HCWs, with a total of 26 reported deaths. Such an elevated number of reported cases may be a direct consequence of the increased attention paid to this category of workers; the overall mortality rate (0.3%) [3] is lower compared to that observed in the general population. However, such a high number of cases cannot be ignored and it is crucial to determine the predictive value of all symptoms that could help to identify and isolate positive workers, thus limiting the spread of infection.

Cases with moderate symptoms are common [4]. The spreading of the virus in winter resulted in even greater difficulties in discriminating between SARS-CoV-2 and other viruses with a similar clinical presentation.

Due to economic and logistic reasons, it is not possible to analyze swabs of every patient and healthcare personnel unless there is a reasonable possibility that they contracted the viral infection. Since severe cases, characterized by acute respiratory distress syndrome, septic shock, metabolic acidosis, and coagulation dysfunction, are relatively easy to recognize, the main challenge is identifying cases with a milder clinical presentation. Having more data regarding the precise predictive value of all minor symptoms could help to filter the high number of subjects suffering from other conditions.

This information, along with laboratory data such as white blood cell count, could lead to a rapid stratification of the population. This would allow the prompt identification of high priority subjects in need of being tested with swabs (analyzed by RT-PCR) [5].

Aside from the well documented respiratory system symptoms, SARS-CoV-2, in some cases, is associated with the development of neurological symptoms such as headache, nausea, dizziness, hypogeusia/ageusia, and hyposmia/anosmia [6]. The role of anosmia and hyposmia as potential COVID-19-related symptoms was already cited by some authors in the scientific literature [7,8,9]. Lechien et al. [10] in a multicenter European study suggested that olfactory and gustatory dysfunctions are considered a clinical presentation of mild-to-moderate forms of COVID-19.

Due to the high number of cases in Italy, it is possible to collect a large amount of data to gather more information about the clinical presentation of this disease, and this could be of great importance for early recognition of the infection in HCWs and to avoid the transmission from HCWs to patients.

As such, we aimed to assess the diagnostic accuracy of symptoms and signs in HCWs with COVID-19 in Italy, looking at which symptoms are most predictive of positive/negative SARS-CoV-2 RT-PCR results among this population.

## 2. Materials and Methods

### 2.1. Study Design and Setting

According to a Regional Decree, the teaching hospital Policlinico Umberto I was classified as an Intervention Hospital for COVID-19. The hospital had 158 beds (including 34 intensive care unit beds) dedicated to the hospitalization of COVID-19 patients. Hence, a specific surveillance system tailored to COVID-19 risk was created by the Health Direction and the Service of Occupational Health for healthcare employee health monitoring. The surveillance procedure established that healthcare employees (HCEs) of all wards with acute respiratory illness (fever and/or at least one symptom of respiratory disease or influenza-like syndrome, e.g., cough, shortness of breath, pharyngodynia, asthenia, myalgia, headaches, thoracic pain, conjunctivitis, rhinitis, diarrhea, anosmia, and ageusia) in close contact with a confirmed or probable COVID-19 case in the last 14 days prior to onset of symptoms would undergo a nasopharyngeal swab for SARS-CoV-2 testing.

Within this context, a case-control study was conducted between 11 and 28 March 2020. In this prospective study, cases were HCEs with an indication for laboratory testing for COVID-19 and laboratory confirmation of SARS-CoV-2 infection.

Controls were HCEs with an indication for laboratory testing for COVID-19 and negative laboratory testing for SARS-CoV-2 infection.

After 28 March, the rules for testing for SARS-CoV-2 were changed in the hospital, and a new surveillance system was set up, testing HCWs in all wards, independent of the presence of symptoms or contact with COVID-19 patients.

The study was approved by the Local Ethics Committee (prot. Number 0566/2020).

### 2.2. Data Collection

A structured interview was administered by trained physicians to each participant before laboratory testing. The following data were collected: socio-demographic characteristics and presence of fever, cough, diarrhea, dyspnea, pharyngodynia, asthenia, headache, anosmia (without obstruction), ageusia, myalgia, arthralgia, thoracic pain, conjunctivitis and rhinitis, using a form for cases of infections from respiratory viruses provided by SERESMI, the Regional Service for Epidemiology, Surveillance and Control of Infectious Diseases (http://www.regione.lazio.it/binary/rl_main/tbl_documenti/All3_69913_24_01_2020.pdf). These symptoms were the only ones that the patients experienced during data collection.

### 2.3. Specimen Collection and Laboratory Test

SARS-CoV-2 was investigated by collecting nasopharyngeal swabs at the Hospital Outpatient Infectious Disease Service. Trained physicians and nurses transferred each specimen to a 3 mL vial with viral transport media (VTM). After collection, specimens were refrigerated at 2–8 °C, unless transported immediately, and refrigerated at 2–8 °C during transport. Laboratory testing was carried out at the Laboratory of Virology in the hospital, located no more than 100 m from the Outpatient Infectious Disease Service. Diagnosis of SARS-CoV-2 infection was performed by RT-PCR (RealStar^®^ SARS-CoV-2 Altona Diagnostic–Germany) after RNA extraction. This procedure for nasopharyngeal swabs was carried out with an automated sample preparation (SP) module using a Versant SP 1.0 Reagents kit (Siemens Healthcare Diagnostics Inc., Tarrytown, NY, USA) [11]. The limit of detection was 1000 cp/mL.

### 2.4. Data Analysis

Data analysis was conducted using a Chi-square test and Student’s *t*-test for differences in categorical and quantitative variables, respectively. Receiver operator curves (ROCs) were used for finding the area under the curve (AUC) for each symptom. The diagnostic accuracy was calculated using sensitivity, specificity, and positive and negative predictive values. Finally, a logistic regression analysis was conducted for calculating the Odds Ratio (OR) and 95% Confidence Interval (95% CI) for each symptom associated to the COVID-19 positivity. This analysis was carried out adjusting the estimate for age and sex.

SPSS 25.0 (Chicago, IL, USA) was used for the analysis. The statistical significance was set at *p* < 0.05.

## 3. Results

We recruited 30 cases and 75 controls. We did not have any excluded patients or invalid or indeterminate results.

In Table 1, some general characteristics of the two groups are presented. There was a higher proportion of men in the group of patients with a positive SARS-CoV-2 RT-PCR test compared to the group with a negative test (53.3 versus 30.7, *p* = 0.03). The average age was not significantly different between the two groups (40.7 versus 43.6 years). The vast majority of cases were HCWs working in medical wards.

In Table 2, the clinical characteristics of cases compared to controls are reported. Notably, while no differences were found between cases and controls for fever and cough symptoms, significant differences were found for dyspnea (*p* = 0.036), headache (*p* = 0.023), and anosmia and ageusia (*p* < 0.001).

The ROC analysis confirmed this interpretation. The diagnostic characteristics (Table 3) show that fever has the highest sensitivity (73.3%), but the best performance for the specificity was related to dyspnea (98.7%), anosmia (93.3%), and ageusia (92%). The highest PPVs were related again to dyspnea (75%), anosmia (73.7%), and ageusia (66.7%), and concerning the combination of symptoms anosmia and ageusia (85.7%) and anosmia and fever (78.6%). Finally, the highest NPVs were related to anosmia (81.4%) and ageusia (79.3%), considering the combination of symptoms anosmia and dyspnea (84.1%) and anosmia and ageusia (80.2%).

The logistic regression models show that anosmia (OR = 14.75; 95% CI: 4.27–50.87), ageusia (OR = 9.18; 95% CI: 2.80–30.15), and headache (OR = 3.92; 95% CI: 1.45–10.56) are significantly associated with SARS-CoV-2 positivity.

Concerning some possible combination of symptoms, anosmia and fever (OR = 19.7), anosmia and other respiratory symptoms (OR = 6.87), anosmia and dyspnea (OR = 18.78), and anosmia and ageusia (OR = 30.65) are significantly associated with SARS-CoV-2 positivity.

Table 4 lists the results by number of symptoms reported at the time of the interview. Total symptoms at that time ranged from zero to six. HCWs reporting three or more symptoms had an increased likelihood of being SARS-CoV-2 positive (OR = 3.18 (95% CI: 1.22–8.28). The odds ratio of a positive RT-PCR increased with additional symptoms and reached 9.93 (95% CI: 0.94–104.4) for six symptoms.

## 4. Discussion

Our case control study is one of the first studies concerning the diagnostic accuracy of symptoms in HCWs with COVID-19 [12]. This study indicates that anosmia and ageusia, among all symptoms, are predictive of COVID-19 positivity. These results partially contrast with those reported in a case control carried out in the general population in Singapore, in which none of the symptoms considered were predictive of positivity [5]. Our results agree with those reported by Vaira et al. [13] in which chemo-sensitive disorders were self-reported by almost 75% of COVID-19 confirmed cases in a multicenter study in Italy; with those reported in HCWs by Villareal et al. [14] in which olfactory or taste dysfunctions were present in 70% of patients and appeared as early symptoms; and with those reported by Brandstetter et al. [15] in which anosmia was present in 51.6% of staff members of a major German children’s and women’s hospital.

Isolated anosmia, in our experience, is not a predictor of COVID-19. It accounts for only 3.3% of all cases, and this puts our study in the lower part of the range reported in a recent review carried out by Mullol et al. [16], according to whom the percentage of smell and/or taste disorders in COVID-19 patients ranges from 5% to 98%, depending on the methodology, country, and study.

Concerning the number of symptoms, our results agree with those reported by Lan et al. [12]. The absence of symptoms or having only one symptom were associated with negative SARS-CoV-2 tests. HCWs reporting three or more symptoms had three-fold greater age- and sex-adjusted odds of having a positive test. These odds showed an increasing trend for each additional symptom reported, with the highest value of OR (almost 10) reached for six symptoms.

Our study highlights the importance of anosmia and ageusia, both frequently reported conditions among our patients, as easy to investigate.

This virus shares many features with other members of the Coronaviridae family like SARS, MERS, and 229E. The members of this family are enveloped, single-stranded RNA viruses with the characteristic shape of a crown surrounding the spherical envelope given by the spikes on the surface. SARS-CoV, MERS, and SARS-CoV-2 are all members of Betacoronavirus [17].

The S protein of the envelope is crucial for the initial attachment and entry into the host cell and is the primary determinant of the cellular tropism of each virus, and a possible target for future treatment [18]. The cell receptor bound by the S protein is different among the various species, and whereas MERS uses dipeptidyl peptidase (DPP4), both SARS-CoV-2 and SARS-CoV share the same receptor: angiotensin converting enzyme (ACE2) [19]. Therefore, we can assume that their cellular tropism could be similar. Based on the high homology in RNA sequences between SARS-CoV-2 and SARS-CoV [20], it is possible to apply some of the information already obtained from SARS-CoV literature, to this new virus. In transgenic mice with a human ACE2 receptor, SARS-CoV showed a high neurotropism. When administered intranasally, infection spreads transneuronally from the olfactory bulb showing a pattern of antigen distribution strongly suggestive of an entry via the olfactory nerve [21]. This may explain why anosmia is one of the first symptoms to appear.

The ACE2 receptor is also expressed in the epithelial cells of the oral mucosa, justifying the possible presence of taste disorder [22]. We think that many answers to the involvement of olfactory and taste pathways will be clarified when histopathological analysis on samples obtained from COVID-19 patients who died are available. 

Our results agree with Clemency et al. [23] concerning the diagnostic characteristics of symptoms (high specificity and negative predictive value) for anosmia and ageusia. We found the level of sensitivity similar to that reported by Brandstetter et al. (51.6% for anosmia) [15] and higher to those reported by Lan et al. (15.6% for anosmia/ageusia) [12] and Wee et al. (22.7% for olfactory and taste disorders) [24]. Regarding specificity concerns, our results are similar to those reported by Lan et al. (97%) [12], Brandstetter et al. (97.6%) [15], and Wee et al. (99.7%) [23]. PPV and NPV in our study were quite high. Nevertheless, these values need careful consideration, since they are strictly related to the prevalence of the disease in the population.

Finally, we noticed that fever and cough had the lowest specificity (less than 50%). In our sample, the proportion of cases with fever (73.3%) and cough (60%) was similar to what Borges do Nascimento et al. found in a systematic review (82% and 61%, respectively) [25].

### Limitations

Possible limitations are related to the relatively small number of HCWs infected, but we recorded all the HCWs in the period considered. Moreover, in the ascertainment of anosmia and ageusia, we did not use chemosensitive evaluation, and this could have led to an underestimation of the symptoms, as underlined by Vaira et al. [13]. We did not perform a re-test for SARS-CoV-2 for negative results, so false a negative for COVID-19 patients could have been an issue. This could have had some consequences, since it cannot be excluded as a risk that some other symptoms did not show a high sensitivity or specificity due to the possibility that there could be false negative patients in the control group.

Concerning other possible biases, the typical bias of case-control studies concerning recall bias was avoided, since data on the presence of symptoms were collected at the same time of swab collection. Moreover, we were not able to test the usefulness to use white blood cell count due to the type of surveillance chosen by the hospital direction. Finally, we need to carefully consider our results since PPV and NPV depend on the prevalence of anosmia and ageusia in a group of subjects and could be different in various hospitals or countries.

## 5. Conclusions

Anosmia and ageusia symptoms are considered as useful indicators for early detection of COVID-19, in addition to the well-established fever, cough, and dyspnea. In particular, these symptoms have a high level of specificity and negative predictive values. According to the study, all HCWs with anosmia and another symptom should be tested for COVID-19, and this could be of particular interest in countries where the use of PCR tests is restricted. In resource-limited settings, this could save time and reduce costs. 

## Figures and Tables

**Table 1:** Characteristics of the study population.

Variables	Controls *n* (%) or Mean (SD)	Cases *n* (%) or Mean (SD)	*p*
*Sex*			0.03
Female	52 (69.3)	14 (46.7)	
Male	23 (30.7)	16 (53.3)	
*Age*	43.6 (12.9)	40.7 (12.9)	0.294
*Type of profession*			0.066
Physicians	24 (32)	12 (40)	
Residents	20 (26.7)	12 (40)	
Nurses	21 (28)	2 (6.7)	
Other healthcare professional	9 (12)	2 (6.7)	
Administrative staff	1 (1.3)	2 (6.7)	
*Ward*			0.004
Medicine	38 (50.7)	26 (86.7)	
Surgery	15 (20)	0 (0)	
Emergency/intensive care	10 (13.3)	1 (3.3)	
Diagnostics	12 (16)	3 (10)	

**Table 2:** Clinical characteristics of the study population.

Variables	Controls	Cases	*p*
*Fever*			0.160
No	31 (41.3%)	8 (26.7%)	
Yes	44 (58.7%)	22 (73.3%)	
*Cough*			0.665
No	35 (46.7%)	12 (40.0%)	
Yes	40 (53.3%)	18 (60.0%)	
*Dyspnea*			0.036
No	74 (98.7%)	27 (90.0%)	
Yes	1 (1.3%)	3 (10.0%)	
*Pharyngodynia*			<0.001
No	47 (62.7%)	29 (96.7%)	
Yes	28 (37.3%)	1 (3.3%)	
*Asthenia*			0.246
No	56 (74.7%)	19 (63.3%)	
Yes	19 (25.3%)	11 (36.7%)	
*Headache*			0.023
No	57 (78.1%)	16 (21.9%)	
Yes	18 (56.3%)	14 (43.8%)	
*Anosmia*			<0.001
No	70 (93.3%)	16 (53.3%)	
Yes	5 (6.7%)	14 (46.7%)	
*Myalgia/arthralgia*			0.838
No	54 (72.0%)	21 (70.0%)	
Yes	21 (28.0%)	9 (30.0%)	
*Diarrhea*			0.741
No	69 (93.0%)	27 (90.0%)	
Yes	6 (7.0%)	3 (10.0%)	
*Thoracic pain*			0.506
No	70 (93.3%)	29 (96.7%)	
Yes	5 (6.7%)	1 (3.3%)	
*Conjunctivitis*			0.190
No	69 (93.0%)	25 (83.3%)	
Yes	6 (7.0%)	5 (16.7%)	
*Rhinitis*			0.345
No	62 (82.7%)	27 (90.0%)	
Yes	13 (17.3%)	3 (10.0%)	
*Ageusia*			<0.001
No	69 (93.0%)	18 (60.0%)	
Yes	6 (7.0%)	12 (40.0%)	
*Anosmia + Fever*			<0.001
No	72 (96%)	19 (63.3%)	
Yes	3 (4%)	11 (36.7%)	
*Anosmia + other respiratory symptoms*			0.001
No	71 (94.7%)	21 (70%)	
Yes	4 (5.3%)	9 (30%)	
*Isolated anosmia*			0.286
No	75 (100%)	29 (96.7)	
Yes	0 (0%)	1 (3.3)	
*Anosmia + Dyspnea*			<0.001
No	69 (92%)	13 (43.3%)	
Yes	6 (8%)	17 (56.7%)	
*Anosmia + Ageusia*			<0.001
No	72 (96%)	18 (60%)	
Yes	3 (4%)	12 (40%)	

**Table 3:** Diagnostic characteristics of symptoms.

Symptom	Sensitivity	Specificity	Positive Predictive Value	Negative Predictive Value	AUC	Adj OR (95% CI)
Fever	73.3	41.3	33.3	79.5	0.573	2.22 (0.83–5.90)
Cough	60.0	46.7	31.0	74.5	0.533	1.30 (0.52–3.24)
Dyspnea	10.0	98.7	75	73.27	0.543	9.83 (0.90–107.22)
Pharyngodynia	3.3	62.7	3.4	61.8	0.330	**0.05 (0.01–0.41)**
Asthenia	36.7	74.7	36.66	74.66	0.557	1.85 (0.71–4.81)
Headache	46.7	76.0	43.75	78.08	0.736	**3.916 (1.45–10.56)**
Anosmia	46.7	93.3	73.7	81.4	0.700	**14.75 (4.27–50.87)**
Myalgia/arthralgia	30.0	72.0	30.0	72.0	0.510	1.02 (0.39–2.70)
Diarrhea	10.0	20.0	33.33	71.88	0.510	1.19 (0.27–5.26)
Thoracic pain	3.3	93.3	16.7	70.7	0.483	0.56 (0.06–5.33)
Conjunctivitis	16.7	20.0	45.45	73.40	0.543	2.40 (0.64–8.94)
Rhinitis	10.0	82.7	18.75	30.34	0.583	0.55 (0.14–2.17)
Ageusia	40.0	92.0	66.7	79.3	0.660	**9.18 (2.80–30.15)**
Anosmia + fever	36.7	96	78.6	79.1	0.663	**19.70 (4.5–86.8)**
Anosmia + other respiratory symptoms	30	94.7	69.2	77.2	0.623	**6.87 (1.87–25.21)**
Isolated anosmia	3.3	100	100	72.1	0.517	not determined
Anosmia + dyspnea	56.7	92	73.9	84.1	0.743	**18.78 (5.66–63.35)**
Anosmia + ageusia	40	97.3	85.7	80.2	0.687	**30.65 (5.78–162.6)**

Adj stands for adjusted. The values in bold are those statistically significant.

**Table 4:** SARS-CoV-2 test results by number of symptoms reported at the time of interview.

Number of Symptoms	Controls	Cases	Adj OR (95% CI)
<2	15 (20%)	4 (13.3%)	0.49 (0.14–1.74)
≥2	60 (80%)	26 (86.7%)	2.03 (0.57–7.17)
≥3	35 (50%)	22 (73.3%)	3.18 (1.22–8.28)
≥4	16 (21.3%)	18 (60%)	5.89 (2.25–15.42)
≥5	4 (5.3%)	6 (20%)	6.04 (1.46–24.93)
6	1 (1.3%)	3 (10%)	9.93 (0.94–104.4)
